# New Cu^+2^ Complexes with N-Sulfonamide Ligands: Potential Antitumor, Antibacterial, and Antioxidant Agents

**DOI:** 10.3390/molecules27103338

**Published:** 2022-05-23

**Authors:** Adriana Corina Hangan, Alexandru Turza, Roxana Liana Lucaciu, Bogdan Sevastre, Emőke Páll, Luminița Simona Oprean, Gheorghe Borodi

**Affiliations:** 1 Department of Inorganic Chemistry, Faculty of Pharmacy, “Iuliu-Hațieganu” University of Medicine and Pharmacy, 400012 Cluj-Napoca, Romania; acomsa6@yahoo.com (A.C.H.); loprean@umfcluj.ro (L.S.O.); 2 National Institute for Research and Development of Isotopic and Molecular Technologies, 400293 Cluj-Napoca, Romania; turzaalex@yahoo.com (A.T.); borodi@itim-cj.ro (G.B.); 3 Department of Pharmaceutical Biochemistry and Clinical Laboratory, Faculty of Pharmacy, “Iuliu-Hațieganu” University of Medicine and Pharmacy, 400012 Cluj-Napoca, Romania; 4 Paraclinic/Clinic Department, Faculty of Veterinary Madicine, University of Agricultural Science and Veterinary Medicine, 400372 Cluj-Napoca, Romania; bogdan.sevastre@usamvcluj.ro (B.S.); emoke.pall@usamvcluj.ro (E.P.)

**Keywords:** sulfonamides, Cu^+2^ complexes, crystal structure, oxidative DNA cleavage, cytotoxicity, SOD-mimetic and antibacterial activities, non-toxic Cu^+2^ complex

## Abstract

Nowadays, the discovery of a new non-toxic metal complex with biological activity represents a very active area of research. Two Cu^+2^ complexes, [Cu(L1)_2_(H_2_O)_3_] (C1) (HL1= N-(5-(4-methylphenyl)-[1,3,4]–thiadiazole–2-yl)-naphtalenesulfonamide) and [Cu(L2)_2_(py)_2_(H_2_O)] (C2) (HL2= N-(5-ethyl-[1,3,4]–thiadiazole–2-yl)-naphtalenesulfonamide), with two new ligands were synthesized. The X-ray crystal structures of the complexes were determined. In both complexes, Cu^+2^ is five-coordinated, forming a CuN_2_O_3_ and CuN_4_O chromophore, respectively. The ligands act as monodentate, coordinating the metal ion through a single N_thiadiazole_ atom; for the C2 complex, the molecules from the reaction medium (pyridine and water) are also involved in the coordination of Cu^+2^. The complexes have a distorted square pyramidal square-planar geometry. The compounds were characterized by FT-IR, electronic EPR spectroscopy, and magnetic methods. The nuclease activity studies confirm the complexes’ capacity to cleave the DNA molecule. Using a xanthine-xanthine oxydase system, the SOD mimetic activity of the complexes was demonstrated. Cytotoxicity studies were carried out on two tumor cell lines (HeLa, WM35) and on a normal cell line (HFL1) using the MTT method, with cisplatin used as a positive control. The antibacterial activity of the complexes was investigated against two Gram-positive and two Gram-negative bacteria, and compared with Amoxicillin and Norfloxacin using the disk diffusion method. Both complexes showed in vitro biological activity but the C2 complex was more active. A lack of in vivo toxicity was demonstrated for the C2 complex by performing hepatic, renal, and hematological studies on Swiss mice.

## 1. Introduction

The development of effective antitumor drugs with high selectivity and low toxicity is currently a major challenge for the scientific community. The success of the Pt(II) ion complex (cisplatin) in the treatment of various cancers places coordinative chemistry among viable antitumor design alternatives [1,2]. Although highly efficacious, treatment with cisplatin is still limited by side effects, inherited resistance, or acquired resistance, which has only partially been eliminated by the introduction of new Pt(II) drugs [3,4].

Current research focuses on the design and synthesis of new complex metal antitumor agents with better biological activity and selectivity, reduced toxicity, and mechanisms of action other than those of platinum compounds, capable of overcoming the unresolved clinical problems of analogue drugs of cisplatin (serious side effects, general toxicity, and resistance) [5,6]. In this context, Cu^+2^ complexes present encouraging prospects [7,8,9,10,11]. The differentiated response of normal and tumor cells to exposure to Cu^+2^ ions is the basis for the development of new copper compounds with antitumor properties. Copper complexes known as “artificial nucleases” have been shown to possess in vitro cytotoxic activity and lower toxicity than platinum derivatives established as antitumor agents [8,9,10]. Many of them are active against tumor cell lines resistant to cisplatin and similar compounds. While platinum compounds bind covalently to DNA nucleobases, copper derivatives interact with DNA by intercalation, electrostatic attraction forces, binding to its “minor cavity”, etc. For most Cu^+2^ complexes reported as DNA molecule cleavage agents, their mode of action is mediated by the formation of reactive oxygen species (ROS) generated either by its reduction to Cu(I), in the presence of reducing agents (e.g., ascorbic acid) or by oxidation of Cu^+2^ ion to Cu^+3^, in the presence of oxidants (e.g., dihydrogen peroxide or molecular oxygen) [9]. Over the past two decades, complex combinations of copper have strengthened their position in drug chemistry, which is evident in the increasing number of compounds that have demonstrated biological activity in in vitro or in vivo testing. In an attempt to mimic the complex DNA–metal complex interactions typical of cisplatin, most studies of the mechanisms of action of copper complexes continue to consider DNA as the primary biological target. In this routine research, the new complexes are thoroughly characterized and preliminarily tested by in vitro screening on several human cancer cell lines and/or interaction studies with the DNA molecule. A critical analysis of the large number of Cu^+2^ complexes synthesized so far makes it possible to extrapolate some basic concepts useful for future research in this field. Therefore, the resulting complex must be stable enough to transfer the metal to the cancer cell without irreversible interactions with physiological entities but unstable enough to allow the metal to interact directly with the binding site after reaching the target substrate [12,13].

From the point of view of coordinative chemistry, the structural variety and the presence in the structure of sulfonamides of N and O donor atoms offer numerous coordination alternatives for metal ions, especially for those of transition metals Cu^+2^, Zn^+2^, Mn^+2^, Ni^+2^, etc. Heterocyclic sulfonamides can function as ligands for the coordination of transition metals through donor atoms in the sulfonamide group (-SO_2_-NH-) and/or other functional groups in the molecule [14,15]. Depending on the synthesis conditions, in the coordination sphere of the metal ion, along with the sulfonamide ligand, solvent molecules and various ions from the reaction medium are frequently found. The resulting complexes often have improved biological activity of ligands due to a superior pharmacological effect, lower toxicity and adverse effects, and increased bioavailability. To date, it has not been possible to obtain sulfonamide metal complexes that meet all the conditions necessary for their introduction into therapy, with the main drawback being the pronounced toxicity. Therefore, the coordinating compounds of sulfonamides remain a topic of great interest, challenging for specialists in the field of coordinative chemistry, bioorganics, biochemistry, and medicine. The experience gained from studies of complex combinations of transition metal ions and heterocyclic ligands (thiazole, triazole, thiadiazole, quinoline) encourages the expansion of chemical and biological research on new heterocyclic sulfonamides and transition metal ion ligands. Given the promising results of studies of nucleic activity on isolated DNA fragments (plasmid pUC18), we have moved to a new stage, namely the synthesis and characterization of new Cu^+2^ complexes and the evaluation of their antiproliferative and antioxidant potential, by conducting studies in vitro and in vivo.

Our scientific research studies are intended to be a step forward in obtaining new complex combinations of Cu^+2^ with N-substituted sulfonamide ligands, with nuclease, SOD-mimetic, cytotoxic, and antibacterial activities, with possible medical applications [16,17,18,19,20]. In this current study, we report the synthesis and crystallographic and physicochemical characterization of two new Cu^+2^ complexes with N-substituted sulfonamides and we demonstrate their “in vitro” nuclease, SOD-mimetic, cytotoxic, and antibacterial activities. In vivo toxicity studies were carried out for the most active in vitro complex.

## 2. Results and Discussion

### 2.1. Crystal Structure Description

Details regarding the crystal structures and refinement of both cooper complexes are given in Table 1.

#### 2.1.1. Crystal Structure of [Cu(L1)_2_(H_2_O)_3_](C1)

**Figure 1:**
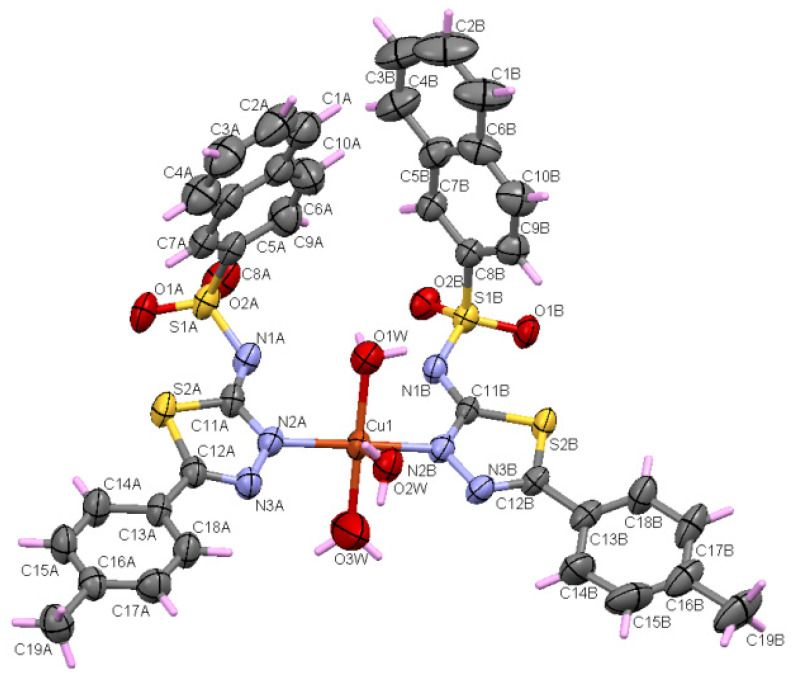
Asymmetric unit of the complex **C1** displaying the atoms as thermal ellipsoids at the 50% probability level.

Using X-ray single crystal diffraction, it was shown that **C1** crystallizes in the centrosymmetric P2_1_/c space group. The asymmetric unit of the **C1** complex consists of two ligands, three water molecules, and one Cu^+2^ ion (Figure 1). It contains a CuN_2_O_3_ entity that adopts a slightly distorted square pyramidal geometry. The Cu^+2^ ion coordinates the three water molecules and the two ligands through two nitrogen atoms (N2A and N2B) that belong to the thiadiazole rings. In this sense, the coordination distances Cu-N_thiadiazole_ (Cu-N2A and Cu-N2B are 2.012 and 2.014 Å) are close to other distances reported in similar copper complexes containing sulfonamides and thiadiazole rings [14,21,22]. On the other hand, water molecules’ coordination distances vary between 1.982 and 2.435 Å and Cu-N distances are almost equal. As an interesting remark, it was found that the coordination of the Cu^+2^ ion takes place through N_thiadiazole_ nitrogen to the detriment of the deprotonated nitrogen of the sulfonamide group. Similar behavior has been reported in other copper complexes [20]. This can be attributed to the charge delocalization of the thiadiazole ring and the sulfonamido group. It can be stated that the L^−^ ligand behaves as monodendate, coordinating through N_thiadiazole_ nitrogen.

The N3-C12 and C11-N2 bond distances are between 1.297 Å and 1.323 Å for both thiadiazole rings and are specific for N-C double bonds (Table S1, Supplementary Material). On the other hand, the N1A-C11A and N1B-C11B distances are shorter than the typical distances for single N-C bonds (1.416 Å) for N (sp^3^) due to the delocalization of charge in the thiadiazole ring. Also due to the delocalization in the thiadiazole ring, the distances N2A-N3A (1.380 Å) and N2B-N3B (1.388 Å) are slightly shorter than the usual N-N distance (1.420 Å). Referring to the C-S distances from the thiadiazole rings (C11A-S2A, C11B-S2B, C12A-S2A, C12B-S2B) (Table S1, Supplementary Material), they are similar to those found in thiophene rings [23], which indicates a π character of the bonds due to the delocalization of the charges. Atoms S1, O1, O2, N1, and C8 adopt a distorted tetrahedral geometry, with the smallest angle for fragment A being 105.0° (N1A-S1A-C8A) and the largest angle being 117.46° (O1A-S1A-O2A) compared to the ideal value of the angle, which is 109.47°. For ligand B, the lowest value is 105.94° for O2B-S1B-C8B and the highest is 117.32° for O1B-S1B-O2B. Table S1 in the Supplementary Material shows that the distances S1-O1 and S1-O2 are typical for double bonds in both A and B fragments. The distances S1A-N1A (1.601 Å) and S1B-N1B (1.602 Å) are consistent with other similar sulfonamides complexes [24]. The S1A-C8A (1.759 Å) and S1B-C8B (1.776 Å) bonds are the longest covalent bonds in the complex. The naphthalene rings in the A and B fragments adopt a planar geometry, with RMSD = 0.007 and 0.015, respectively. The two thiadiazole rings adopt a planar geometry, with an RMSD equal to 0.009 and 0.011, and form an angle of 10.49° between the two planes.

**Figure 2:**
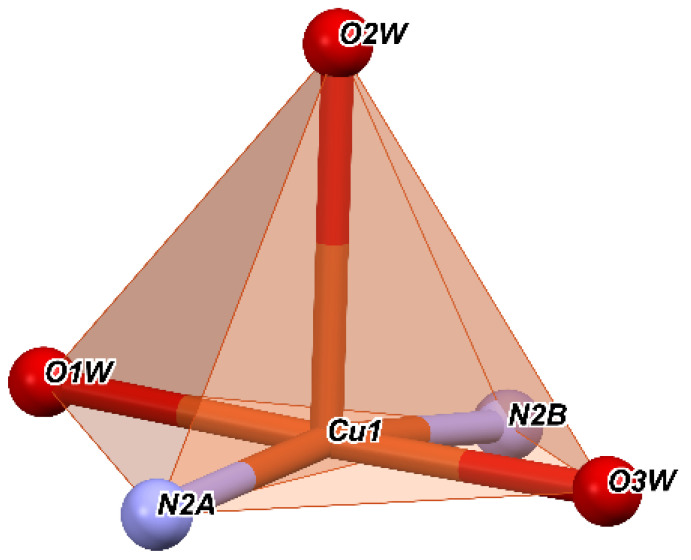
Coordination polyhedron of complex **C1**.

If we consider that the nitrogen atoms and the water molecules O1W and O3W are located in a basal plane and O2W is considered outside this plane, then we obtain a square pyramid with the top in O2W (Figure 2), with Cu^+2^ being located roughly in the center of the pyramidal base. Then, the angles of the equatorial plane are close to 90° and vary between 87.89° and 92.20°. Moreover, the angles determined by the atom at the top of the pyramid (O2W), the Cu ion, and the atoms at the base have values between 86.61° and 95.09°.

It suggests that the nitrogen atoms together with the oxygen ones involved in the Cu^+2^ coordination form a polyhedron with distorted square pyramidal geometry. The values of the trigonality index τ = 0.02 and tetragonal distortion index T^5^ = 0.87 indicate a distorted square pyramidal geometry.

**Figure 3:**
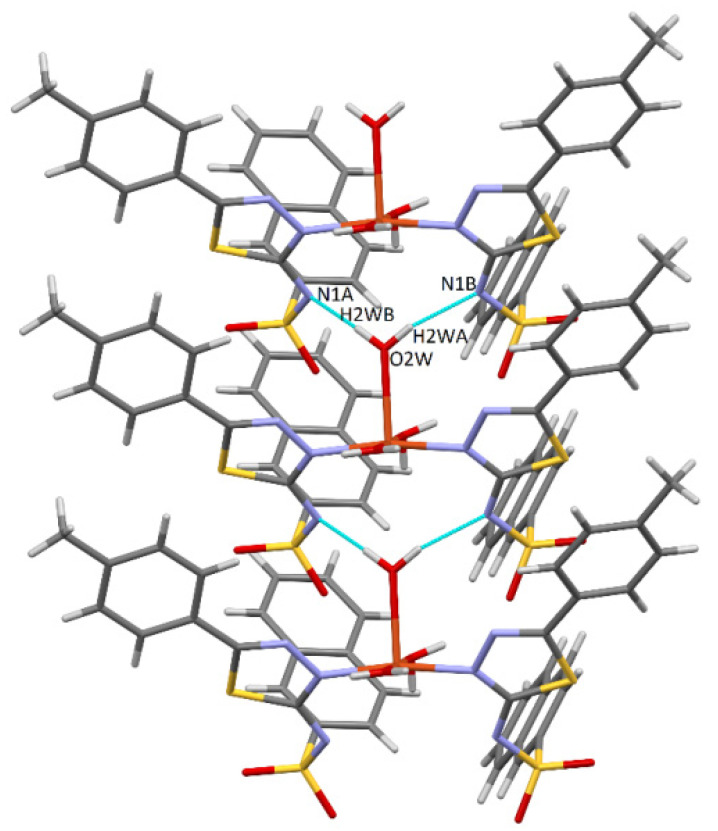
Bifurcated O-H···N hydrogen bonds in complex **C1**.

**Figure 4:**
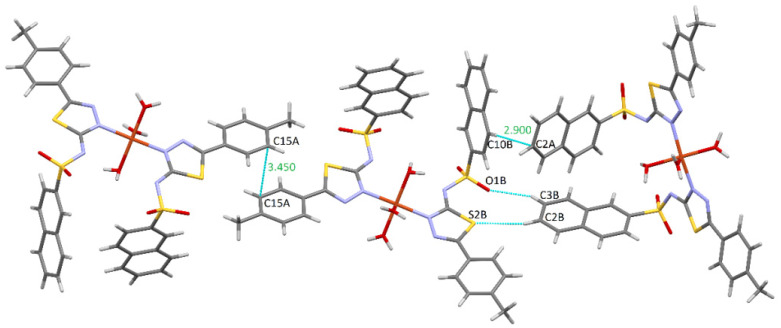
π···π, C-H···π, and other weak van der Waals interactions in complex **C1**.

The cohesion of the complex within the crystal lattice is sustained by the bifurcated hydrogen bonds O-H···N between the oxygen of the O2W water and both deprotonated sulfonamide nitrogens of the two ligands (N2A and N2B) (Figure 3). Furthermore, the water molecule O1W also plays a role through the O-H···O hydrogen bond, being connected to the O2B sulfonamido oxygen of an adjacent ligand. It is also worth noting the existence of π···π interactions with a separation distance of 3.45 Å between two phenyl rings of the same ligand, and C-H···π contacts (involving naphthalene rings, 2.90 Å) contribute to the stability as well. Other weak intermolecular interactions (C2B-H···S2B and C3B-H···O1B) that display d (H···A) shorter than the sum of van der Walls radii are shown in Figure 4.

**Figure 5:**
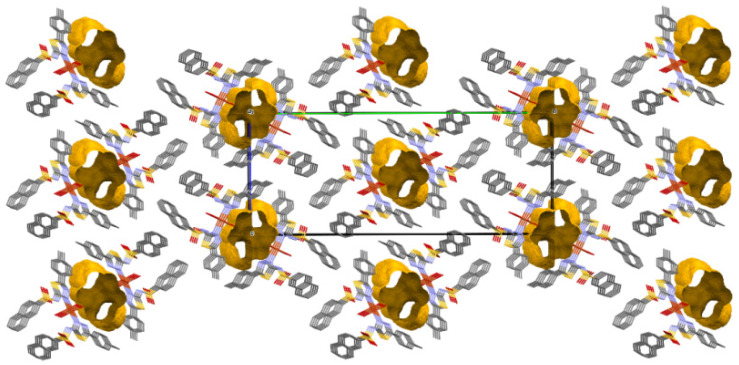
Structural voids in complex **C1**.

An interesting feature of this complex is the existence of structural voids. A void is a region with a radius of minimum 1.2 Å, whose volume does not contain any van der Waals spheres of some neighboring atoms. Knowing that most structures possess a packing index of 64%, this complex contains donut-like voids and displays an index of 58.7%. For this reason, the O3W hydrogens of the water molecule do not participate in the formation of intermolecular hydrogen bonding. A slightly tilted packing perspective along the *oa*-axis is presented in Figure 5, with the voids located in the center and on the *ob* edges of the unit cell, respectively. In general, the existence of structural voids offers the possibility of incorporating solvents into the crystal structure [25].

#### 2.1.2. Crystal Structure of [Cu(L2)_2_(py)_2_(H_2_O)] (C2)

**Figure 6:**
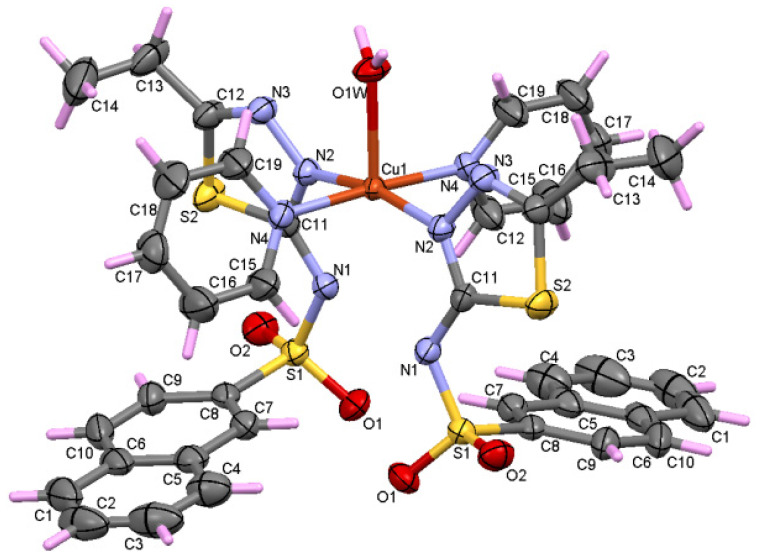
Molecular perspective of complex **C2** displaying the atoms as thermal ellipsoids at the 50% probability level.

The **C2** complex (Figure 6) crystallizes in the monoclinic system, the C2/c centrosymmetric space group. The asymmetric unit consists of a ligand (L^−^), a pyridine ring from the reaction medium, a Cu^+2^ ion, and a water molecule. Both the Cu ion and water participate in the asymmetric unit with half because both are located in a special position (2-fold rotation axis). The Cu ion is coordinated by four nitrogen atoms and the oxygen of the water, forming a CuN_4_O entity in the form of a slightly distorted square pyramid. The ligand coordinates to Cu via the nitrogen atom (N2) with a Cu-N_thiadiazole_ distance of 1.987 Å. The pyridine ring also coordinates through nitrogen to the Cu ion with a Cu-N_pyridine_ distance of 2.064 Å. The coordination distance between Cu^+2^ and the water molecule is 2.285 Å. As in the CG-50 complex, the N_thiadiazole_ nitrogen that participates in coordination is not the deprotonated one of the sulfonamide group, probably due to the delocalization within the thiadiazole ring.

The significant distances and angles of the **C2** complex are shown in Table S2 in the Supplementary Material. The N2-C11 bond distance (1.323 Å) has a normal value for the thiadiazole ring, while N3-C12 is at the lower limit for such covalent bonds. As in the **C1** complex, the N2-N3 distance (1.386 Å) is slightly shorter than the typical distance (1.420 Å). Due to the charge delocalization, the C-S distances are similar to those of the **C1** complex, indicating a π character of the bonds. Similar to the **C1** complex, the S1-O1-O2-N1-C8 atoms have a distorted tetrahedral geometry, with the smallest angle being 106.04° (N1-S1-C8) and the largest N1-S1-O2 (116.71°); the ideal value is 109.47°. The distances S1-N1(1.59 Å) and S1-C8 (1.77 Å) are similar to those of the **C1** complex and the distances S-O are typical for double bonds. Compared to the **C1** complex, which has two independent naphthalene fragments, **C2** has a single fragment because the second is generated by symmetry and has an RMSD = 0.010, indicating a small deviation from planar geometry.

**Figure 7:**
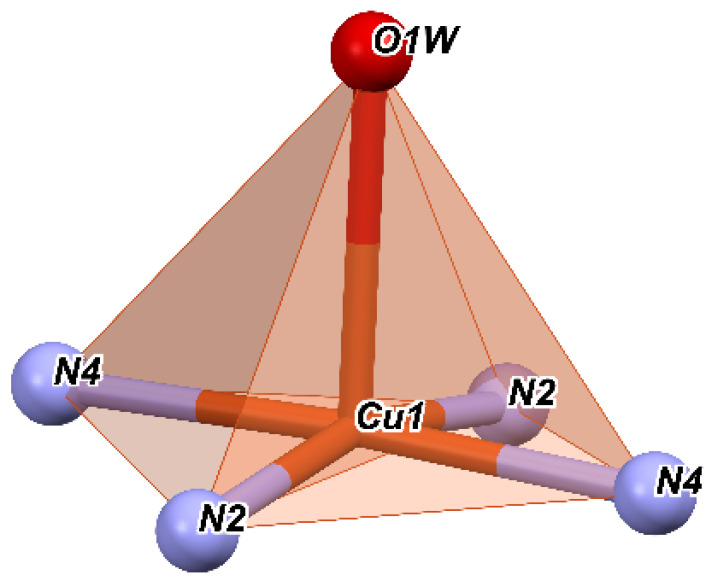
Coordination polyhedron of complex **C2**.

The slightly distorted coordination polyhedron in **C2** is shown in Figure 7. Nitrogen atoms form the base of the pyramid, and the oxygen of the water molecule is the top of the pyramid. The angles of the equatorial plane are close to 90° and have values of 86.81 and 92.79°; the other two angles are identical to the first and generated by symmetry. The tetragonality parameter is T^5^ = 0.88 and the trigonality τ = 0.07.

**Figure 8:**
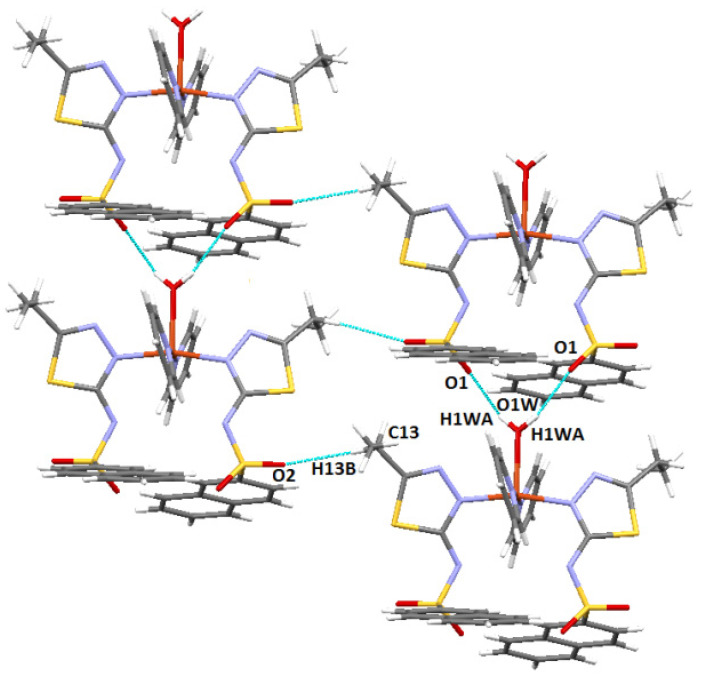
Intermolecular interactions in complex **C2**.

The stability of the complex is ensured by bifurcated O-H···O and C-H···O hydrogen bonds. In the *ob* direction, the water molecule connects two sulfonamide groups (O1W-H···O1), forming an infinite chain along this direction. These chains are connected to each other by the C13-H···O2 interaction, with the oxygen belonging to the sulfonamide group (Figure 8).

### 2.2. Powder X-ray Diffraction Analysis

**Figure 9:**
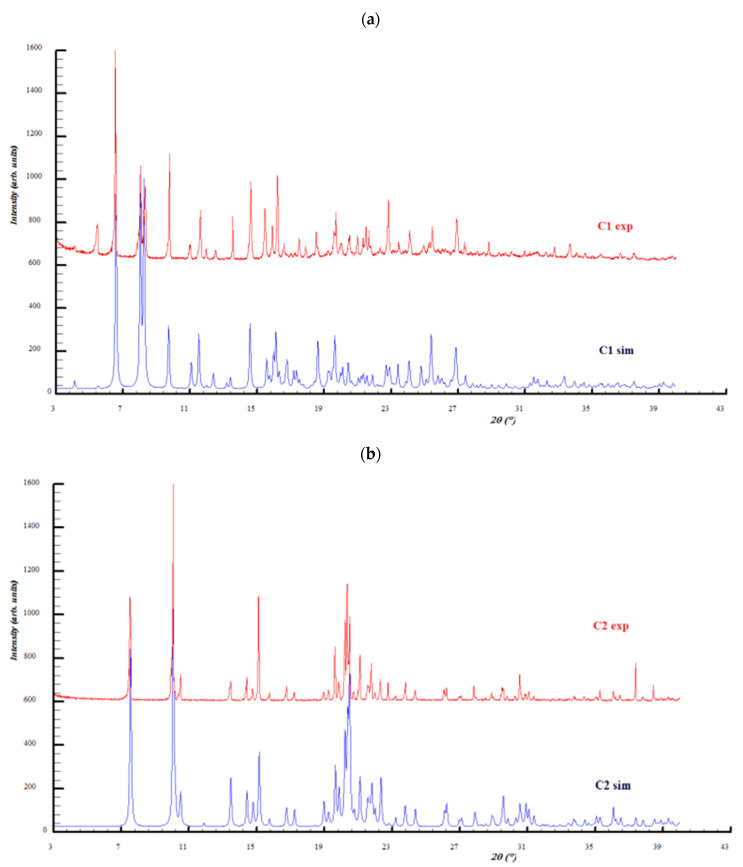
X-ray diffraction patterns’ comparison of the experimental (exp) with the simulated (sim) investigated copper complexes: **C1** (**a**) and **C2** (**b**).

Based on the atomic coordinates in the unit cells, powder X-ray diffraction patterns were simulated by Mercury software [26] and compared with the experimental ones (Figure 9). The experimental powder X-ray diffraction patterns of both complexes indicate a good match with the calculated patterns from the single crystal structures. As a conclusion, the complexes samples are characterized by good purity and structural homogeneity, with the analyzed single crystals being representative of the entire sample where they come from. Slight differences between the simulated and experimental powder diffraction intensities are due to the preferred orientations of the crystallites. The most obvious appears for the C1 complex at 2Ɵ = 5.5°, which appears much more prominent in the experimental pattern and is related to the (0 1 1) crystallographic plane.

### 2.3. Hirshfeld Surfaces and Fingerprint Plots Analysis

Hirshfeld surfaces can be understood as follows: the strong interactions with the intermolecular distances shorter than the sum of van der Waals radii are mapped in red [27], intermolecular contacts with distances longer than the sum of van der Waals radii are mapped in blue, while the distances equal to the sum of van der Walls radii are seen as white areas. The 2D fingerprint plot is a representation of all (d_i_, d_e_) pairs on the Hirshfeld, where d_i_ represents the interior distance between the nucleus and the Hirshfeld surface and d_e_ is the exterior distance from the surface to the neighboring atom. The color code of the fingerprint plots is as follows: the red regions represent a high density of such pairs, green represents a moderate density, and blue indicates a low density of pairs [28].

**Figure 10:**
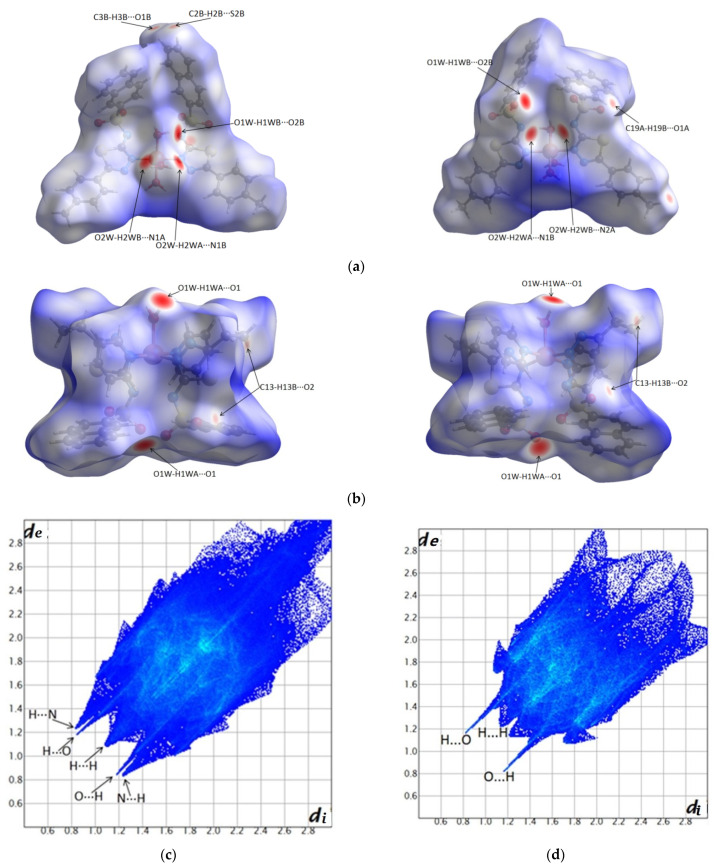
Front and back perspectives of the Hirshfeld surfaces mapped with d_norm_ displaying the intermolecular contacts referred to in Table S3 in the Supplementary Material: **C1** (**a**) and **C2** (**b**). Surfaces are represented with the color scale as follows: −0.44 (red) to 2.9 (blue) for **C1** and −1.27 (red) to 1.78 (blue) for **C2**; fingerprint plots of **C1** (**c**) and **C2** (**d**).

Figure 10 presents the Hirshfeld surfaces of the **C1** complex (a), **C2** complex (b), and the corresponding fingerprint diagrams for **C1** in (c) and **C2** in (d). It is observed that the strongest interactions of the complexes with the neighboring molecules are manifested through hydrogen bonds.

The geometries of the hydrogen bonds and other interactions are detailed in Table S3 in the Supplementary Material, which are further represented by arrows on the Hirshfeld surfaces (Figure 10) (see Hirshfeld surfaces and fingerprint plots analysis section).

Corroborating the Hirshfeld surfaces with the corresponding fingerprint plot diagrams, it is observed that in the case of the **C1** complex, there are strong O-H···O hydrogen bonds (between water and sulfonamide groups) and O-H···N (between water and nitrogen deprotonated sulfonamide).

It is observed that the fingerprint diagram highlights the existence of pairs of distances di and d_e_ pairs with distances that extend up to 3 Å and are correlated with the existence of structural voids.

In the case of the **C2** complex, the red spots on the Hirshfeld diagram and the spikes on the fingerprint plot indicate strong O-H···O interactions between water and sulfonamide.

### 2.4. Spectroscopic and Magnetic Properties

Comparison of the IR spectra of metal complexes with the IR spectra of the corresponding uncoordinated ligands indicates band displacements characteristic of the groups involved in coordination: ν(thiadiazole), ν_s_(SO_2_), ν_as_(SO_2_), and ν(S-N). In the coordination of the Cu^+2^ ion, bands characteristic of the molecules of the reaction medium were also identified.

An important change occurs in the bands characteristic of the valence vibrations of the thiadiazole ring, ν(thiadiazole). The shift of the band to lower frequencies in the IR spectrum of the complex than that of the ligand demonstrates the involvement of the thiadiazole heterocycle through the N atom in the coordination of the Cu^+2^ ion, and the deprotonation of nitrogen from the sulfonamide group N of the thiadiazole nucleus and those belonging to the group—N^(−)^–SO_2−_ [29].

At the level of the sulfonamide group, changes are observed at the level of symmetrical valence vibrations ν_s_(SO_2_) and asymmetric ν_as_(SO_2_) of the S = O bond by moving the bands towards lower frequencies of the IR spectrum in the case of the complex. The band attributed to the vibrations of the S-N, ν(S-N) link in the same group undergoes shifts to higher or lower frequencies of the spectrum. These changes at the level of the sulfonamide group are attributed to the deprotonation of the nitrogen within it, which also determines the appearance of a weak conjugation phenomenon between the three N, S, and O atoms of the group [30,31]. Thus, in the spectrum of complex C2, there are bands assigned to the pyridine molecule that appear at frequencies between 995 and 1073 cm^−1^ [32], but at the same time, there are bands with different spectral positions located at 1047, 1449, and 1492 cm^−1^. The displacement of the bands can be attributed to changes in the vibrational frequency characteristic of the ν(C-C) bond in the pyridine molecule due to its involvement in the coordination of the Cu^+2^ ion through the nyridinic atom. The features of the IR spectrum of the complex are similar to those reported for the other copper *N*-sulfonamide derivatives [33,34,35] and demonstrate the existence of new linkages within the complex (Figures S1 and S2 Supplementary Materials).

The solid electronic spectra of both complexes display a band at 418 (**C1**) and 423 nm (**C2**) assigned to an LMCT transition. The complex (**C1)** exhibits a d-d band at 598 nm and the complex (**C2)** shows a d-d band at 587 nm [29]. This pattern, characteristic for a distorted square-pyramidal, agrees well with the crystallographic data. The UV–Vis spectra of complexes C1 and C2 in DMF–cacodylate buffer (0.1 M, pH 6.0, 39:1) shows a d–d band at 595 (ε = 100 M^−1^∙cm^−1^) and 580 nm (ε = 94 M^−1^∙cm^−1^), respectively. The similarity of the solid liquid electronic spectra indicates that the chromophores of both complexes remain in solution (Figures S3 and S4 Supplementary Materials).

The polycrystalline X-band EPR spectra of both complexes (**C1** and **C2**) are axial. The EPR parameters, obtained by simulation, are g_║_ = 2.32, g_┴_ = 2.074, and A_║_ = 144 × 10^–4^ cm^–1^ for complex (**C1)** and g_║_ = 2.23, g_┴_ = 2.024, and A_║_ = 156 × 10^–4^ cm^–1^ for complex (**C2**) [36]. According to the Bertini [37] classification, the value of A_║_ for complex (C1) and (**C2**) is correlated with the geometry of the complex. Thus, A_║_ values between 130 and 160 cm^–1^ correspond to a square pyramidal or distorted trigonal bipyramidal geometry. Since g|| > g_┴_, the unpaired electron is in the d_x_^2^_−y_^2^ or d_xy_ orbital (Figures S5 and S6 Supplementary Materials).

The room temperature measurements of the magnetic moments of complex (**C1**) (μ_eff_ = 1.73 MB) and complex (**C2**) (μ_eff_ = 1.82 MB) are consistent with the presence of a single unpaired electron.

### 2.5. In Vitro Biological Assays

#### 2.5.1. DNA Cleavage

The activity of the complexes as chemical nucleases was studied using supercoiled pUC18 DNA in cacodylate buffer (0.1M, pH 6.0) in the presence of H_2_O_2_/ascorbic acid, with a 3.0-fold excess relative to the complex concentration. The efficiency of the complexes was compared with that of both copper sulfate and bis(o-phenanthroline) Cu^+2^ complex under the same reaction conditions. The results, as shown in Figure S7 in the Supplementary Material, show that both complexes exhibit nuclease activity and are more effective at higher concentrations.

The two complexes have the ability to destroy the DNA molecule, with this being done gradually, with the appearance of the circular shape at a complex concentration of 15 (**C1**) or 10 μM (**C2**), and then a linear shape of the DNA molecule at a concentration of 20 and 15 μM, respectively. At these last two concentrations, the circular and linear shape coexist, while at the concentration of 15 μM, the presence is observed besides the circular and the helical shape. Initially, cleavage occurs at a single point in a chain in the structure of DNA with the appearance of the circular shape, and later at another point in that chain, which will cause the appearance of the linear shape.

The copper salt, CuSO_4_, at a concentration of 15 μM destroys the DNA molecule, with the appearance of the circular shape, a shape that is maintained at higher concentrations (15 and 20 μM). Comparing the nuclease activity of CuSO_4_ at the same concentrations with those of the complexes, it is observed that the latter has a higher nuclease activity.

The complexes are structurally similar, differing only in the aryl residue from the sulfonamide ligand or the number of the aromatic rings in their structures. The geometry of the molecule determined by the spatial arrangement of ligands with different substituents as planarity influences the ability of the complex molecule to intercalate between the nitrogenous base pairs in the structure of DNA molecules. The more aromatic rings the ligands have in the structure, the easier this “approach” of the Cu^+2^ complex to the target molecule is [18,19,20]. The ability to destroy the DNA molecule is superior to the **C2** complex compared to **C1**. This can be explained by the fact that it is present in the structure in addition to the two molecules of the sulfonamide ligand and the two molecules of pyridine. These four molecules with planar aromatic rings cause the complex molecule to move closer to the nucleic acid, by intercalating between neighboring base pairs in the DNA strands. Both complexes present an inferior capacity to destroy the DNA molecule than the Cu(phen)_2_ complex consecrated as the first “chemical nuclease”.

The coordination of the Cu^+2^ ion with the sulfonamide deprotonated ligand (L^−^) facilitates the destruction of the DNA molecule. Due to the plane aromatic rings in its structure, the sulfonamide allows the complex molecule to be intercalated between base pairs in the DNA chains. This phenomenon is followed by the destruction of the nucleic acid, caused by the production of reactive species of oxygen in its close vicinity [38,39].

The nuclease activity of the complexes **C1** and **C2** was studied in the presence of certain inhibiting agents: DMSO, *t*-butyl alcohol, distamycin, sodium azide, 2,2,6,6-tetramethyl-4-piperidone, superoxide dismutase, and neocuproine, in an attempt to determine the ROS involved in the degradation process of the DNA molecule. We chose a concentration of 10 μM for the complexes and a concentration 30 times higher for the reducing agents ascorbic acid/H_2_O_2_. The incubation period of the samples was 1 h, at 37 °C. The resulting electropherograms are presented in Figure S8 in the Supplementary Material.

The most common mechanisms in the series of Cu^+2^ complexes capable of destroying the DNA molecule are oxidative or hydrolytic degradation. The first mechanism involves the existence of oxidation-reduction processes with the formation of ROS, capable of destroying the helical structure of the DNA molecule, while the second mechanism is an oxygen-independent, non-oxidative process [13,22,39].

To elucidate the mechanism of action as potential “chemical nucleases” of synthesized Cu^+2^ complexes, their nuclease activity was studied in the presence of possible inhibitory agents of different reactive oxygen species (DMSO, SOD, sodium azide (NaN_3_), neocuproin). These studies identified groups or radicals involved in DNA destruction. All the inhibitory agents used more or less diminished the nuclease activity of the complexes, which demonstrates a possible mechanism of oxidative degeneration in their case.

The presence of DMSO (lines 5 and 10) causes a decrease in the nuclease activity of the complex (the helical shape of the nucleic acid coexists with the circular one), which demonstrates the involvement of HO• radicals in the DNA degradation process.

In the presence of SOD (line 6 and 11), there is a decrease in the nuclease activity of the complex, which shows that the superoxide radical anions O_2_•^−^ are involved in the destruction of the nucleic acid molecule.

As for the ability of distamycin to compete with the complex molecule for interaction with the nitrogenous bases of the helical chain of DNA, it is quite small because the nuclease activity of the complex is not diminished (line 7 and 12). The interaction of the complexes with the nucleic acid molecule appears to be different from that of distamycin.

Neocuproin also causes a decrease in the complex’s nuclease activity (line 8 and 13), following the formation of a more stable combination with the Cu^+1^ ion, resulting from the reduction of the Cu^+2^ ion in the complex. Thus, the Cu^+1^ ion that is blocked can no longer participate in the subsequent reactions of generating active radicals responsible for the destruction of DNA.

Sodium azide has little effect on the ability of the complex to destroy the nucleic acid molecule in **C1** and **C2** complexes (lines 9 and 14). Thus, the ^1^O_2_ singlet oxygen contributes to a small extent to DNA degradation, probably forming as an intermediate and being rapidly inactivated by other species in the reaction medium.

In conclusion, the active oxygen species resulting during the mechanism of action of the complexes are HO• and O_2_•^−^. As a result, for the studied complexes, the proposed mechanism involves Fenton-type or Haber–Wiess-type reactions [40,41].

**Figure 11:**
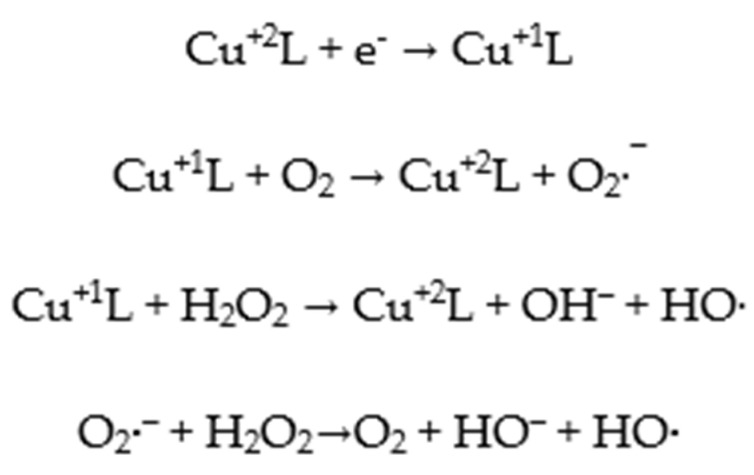
Possible ROS reactions in **C1** and **C2** complexes.

The process of destroying the DNA molecule by synthesized Cu^+2^ complexes comprises the following steps:

1. Interaction of ligands (deprotonated sulfonamide L^-^ and pyridine) with nitrogenous bases in the DNA molecule by π stacking and/or hydrogen bonding.
2. Reduction of Cu^+2^ to Cu^+1^ ion within the complex molecule.
3. Fenton or Haber–Wiess reactions with active radical production (HO• and O_2_^−^), which will break the helical chains in the DNA structure in one place, with the appearance of the circular shape, or in two points of the same chain, with the appearance of the linear one. A possible pathway for ROS generation involved in the degradation of DNA molecules is outlined as follows (Figure 11):

#### 2.5.2. SOD Mimetic Activity

Since the discovery of the functionality of the enzyme SOD, intensive efforts have been made to develop the enzyme as a therapeutic agent for the treatment of diseases that are associated with oxidative stress. For several reasons, such as the size and instability of the SOD enzyme, these attempts to develop the natural enzyme for clinical use have been largely unsuccessful. A great deal of interest represents the development of therapeutic SOD mimetics for the scavenging of superoxide (O_2_*^•^*^−^), which is a precursor to reactive oxygen and nitrogen species that are known to contribute to oxidative stress. For this reason, several metal complexes have been shown to exhibit significant SOD mimetic activities [42].

The SOD-like activities of complexes **C1** and **C2** were determined by an indirect method using the xanthine/xanthine oxidase system as the source of superoxide radicals. Complex **C2** was more potent than **C1**; the IC_50_ values were 0.174 and 0.283 μM. The IC_50_ value for native Cu_2_Zn_2_SOD is 0.006 μΜ. Thus, the IC_50_ values for both complexes are higher than the IC_50_ value for native Cu_2_Zn_2_SOD but are similar to those of complexes with related sulfonamide ligands presented in the literature [43,44,45,46].

The mechanism believed to be operating in the naturally occurring superoxide dismutases involves one-electron reduction of the metal ion of the active center by superoxide followed by reoxidation of the reduced metal ion by a second superoxide anion. Metal complexes that can undergo such redox cycling are likely to function as superoxide scavengers. It is assumed that electron transfer between the metal center and superoxide anion radicals occurs by direct binding [47].

#### 2.5.3. Cell Culture and Cytotoxicity Assays

Three cell lines were used to determine the cytotoxicity of the complexes, a human cervical carcinoma line (HeLa), a human radical growth phase melanoma cell line (WM35), and a normal fibroblastic epithelial cell line (HFL1) using the MTT method. The cells were exposed to C1 and C2 complexes in different concentrations (50, 25, 12.5, 2, and 0.2 µM) at 24, 48, and 72 h. As positive contro, cisplatin was used. The results of the cytotoxicity assays are presented in Table 2.

On both tumor cell lines, the C2 complex was more potent than cisplatin, with the IC_50_ values being about three times lower for HeLa cell lines and about two times lower for WM35 cell lines, at 24 h. For the normal fibroblastic epithelial cell line, the C2 complex is less cytotoxic than cisplatin (Table 2).

C1 complex is less cytotoxic than cisplatin on both tumor cell lines, so is less effective. Concerning the cytotoxicity on the HFL1 cell line, the C1 complex is less cytotoxic than cisplatin and the C2 complex, so is less toxic.

The cytotoxicity of both complexes on both tumor cell lines increases with the time of exposure (from 24 h up to 72 h), so a time-dependent effect could be established.

HL1 and HL2, the free ligands, showed no significant cytotoxicity. This means that the cyytotoxicity is given by the complexes and that the chelation of the ligands with the copper ion is essential for the cytotoxicity of the complexes.

As a conclusion, we synthesized a copper complex, C2, which presents better cytotoxicity on the HeLa and WM35 cell lines than cisplatin, with less toxicity on normal fibroblastic cells than cisplatin. The results are consistent with our DNA cleavage studies.

The capacity of the synthesized copper complexes to destroy the DNA molecule depends on the type and geometry of the ligand participating in the coordination of the Cu^+2^ ion. The ligands have two important roles in the biological activity of the complexes: they influence the reactivity of the Cu^+2^ metal and interact with DNA. The synthesized sulfonamides used as ligands in the obtained complexes increase the reactivity of the metallic ion and the biological activity of the complex compared to simple Cu^+2^ salts. The presence of aromatic rings, such as benzene, toluene, and naphthalene, in the structure of the sulfonamides is probably the main variable determining their capacity to destroy DNA. Their complexes have a higher capacity to destroy DNA than the non-coordinated sulfonamide ligand. These plane aromatic rings allow the complex molecule to come closer to DNA through intercalation between neighboring base pairs of the DNA chains, and then link with them through π-stacking bonds. The biological activity of the complexes depends greatly on the type of ligand used, having a benzene, toluene, or naphthalene ring. There differences among these compounds are due to possible steric hindrances, which can appear in those that contain a toluene or naphthalene ring. In the case of all the synthesized complexes, the ligand participates in the coordination through a single N_thiadiazole_ atom (and is thus monodentate). The geometry of the complexes is similar (more or less distorted square pyramidal). Thus, the differences among the biological activity of the complexes are due to the participation of the second type of ligand in the coordination of the metallic ion (pyridine, ethylenediamine, phenantroline).

The presence of phenantroline as a ligand for the synthesized complexes allows a biological activity at much smaller concentrations than in the case of the compounds that have a similar structure and geometry but have no phenantroline. The literature indicates the efficiency with which the Cu^+2^ or Cu^+1^ complexes, such as Cu-phen or Cu(phen)_2_, interact with the DNA molecule through the secondary sites, probably through intercalation. In the presence of a reducing agent such as hydrogen peroxide, these Cu^+2^ complexes with phenantroline start an oxidative attack on C-1’ and C-4’ of the 2-deoxyribose moiety, leading to the destruction of DNA [17,18,19,20].

#### 2.5.4. Evaluation of Antibacterial Activity

The disk diffusion method was used to determine the antibacterial activity of the **C1** and **C2** copper complexes on four reference microbial strains, two Gram-positive (*Staphylococcus aureus* and *Bacillus cerreus*) and two Gram-negative (*Escherichia coli* and *Pseudomonas aeruginosa*) strains. The obtained results are presented in Table 3.

By comparing the diameters of the bacterial growth inhibition areas, the antibacterial activity of the two copper complexes was demonstrated. The diameters of the inhibition areas were lower for the copper complexes as compared with the reference antibiotics Amoxicillin and Norfloxacin; therefore, both **C1** and **C2** complexes have a weaker antibacterial activity. The exception is the fact that both complexes show antibacterial activity on *Pseudomonas aeruginosa*, which is resistant to Amoxicillin exposure. However, the antibacterial activity of copper complexes on *Pseudomonas aeruginosa* is lower than the antibacterial activity of Norfloxacin. The antibacterial activity of the **C2** complex is slightly superior to that of the **C1** complex on all four bacterial strains.

The sensitivity of the Gram-positive and Gram-negative bacterial strains to the two copper complexes that exhibited antibacterial activity using the disk diffusion method was established by determining their minimum inhibitory concentration (MIC). The values of MIC, defined as the lowest concentration of antimicrobial complex that prevents visible growth of germs, are presented in Table S4 in the Supplementary Material.

All results obtained using the disk diffusion method were concordant with the results obtained in terms of MIC.

Identification of the acquired antibiotic resistance of bacteria and the discovery of new antibacterial compounds is an important goal.

### 2.6. In Vivo Toxicity Study of C2 Complex

We chose to study the in vivo subacute toxicity of the **C2** complex because it showed better in vitro biological activity than C1. This study was carried out for a period of 2 weeks on 10 adult Swiss mice, with 5 males and 5 females.

A slight body weight gain was observed for all mice, but there were no significant changes. Tables S5–S8 in the Supplementary Materials show that the biochemical and hematological parameters were within the normal limits [48]. This demonstrates the lack of hepatic, renal, and hematological toxicity of the C2 complex.

For the kidney and liver, the histological analyses showed a normal structure. The renal glomerulus is well defined in the kidney cortex. Counted tubes present intact cells, with the spherical core located centrally. The conjunctivo-vascular tissue in the cortical stroma is poorly represented. The hepatocytes are separated by sinus capillaries, bile ducts, and supportive tissue.

Remack’s cellular cords are well-acclaimed, intralobular support tissue is represented by connective tissue, and collagen fibers and reticulin are present in a small amount (Figure S9, Supplementary Material).

Both biochemical and histological analyses indicate normal renal and hepatic function. The mice did not undergo clinical changes throughout the study, the biochemical/hematological parameters were within the normal limits for all the studied mice, and all the mice survived until the end of the study. Therefore, the C2 complex is safe.

## 3. Materials and Methods

Copper sulfate pentahydrate, copper nitrate trihydrate, methanol, ammonium hydroxide, and pyridine were purchased from Fluka and Merck chemical companies and were used without further purification.

Elemental analyses (C, N, H, S) were performed with a Vario EL analyser. IR spectra were recorded with a Jasco FT-IR-4100 spectrophotometer using diffuse reflectance of incident radiation focused on a sample, in the 4000–450 cm^–1^ range. All melting points were determined in open capillaries with an Electrothermal IA 9100 apparatus and were uncorrected. The ^1^H NMR spectrum of the ligand was recorded at room temperature using DMSO-d6 as solvent in 5 mm tubes on a Bruker AM 300 NMR spectrometer equipped with a dual ^1^H (multinuclear) head operating at 300 MHz for protons. The fast ion bombardment (FAB) mass spectrum of the ligand was obtained on a VG Autospec spectrometer using m-nitrobenzene as a solvent. Diffuse reflectance spectra and UV-Vis spectra of the complexes were recorded on a Jasco V-550 spectrophotometer. Magnetic susceptibility was measured at room temperature with a Faraday MSB-MKI balance. Hg[Co(NCS)_4_] was used as the susceptibility standard. The electronic paramagnetic resonance (EPR) spectrum was recorded at room temperature with a Bruker ELEXSYS spectrometer operating at the X-band frequency [49].

The sulfonamides HL1 and HL2 were prepared by reacting 2-amino-5-(4-methylphenyl)-[1,3,4]-thiadiazole or 2-amino-5-ethyl-[1,3,4]-thiadiazole with naphtalenesulfochloride, in accordance with the method described elsewhere [16,50]. The elemental chemical analysis data are in agreement with the formulae C_19_H_15_N_3_O_2_S_2_ and C_14_H_13_N_3_O_2_S_2_, respectively.

### 3.1. Synthesis of the Complex [Cu(N-(5-(4-Methylphenyl)-[1,3,4]-Thiadiazole-2-yl) Naphtalenesulfonamidate)_2_(H_2_O)_3_] (C1)

A solution containing 1 mmol copper nitrate trihydrate in 10 mL methanol was added dropwise to 1 mmol of the sulfonamide ligand (HL1) dissolved in 20 mL methanol, under continuous stirring. The resulting solution was stirred at room temperature for two hours and left to stand at room temperature After one week, by slow evaporation of the solvent, green crystals suitable for X-ray diffraction were obtained.

Anal. Calcd for **(C1)** C_38_H_34_CuN_6_S_4_O_7_ (MW = 878.50 g ∙ mol^–1^): C, 51.90; H, 3.87; N, 9.56; S, 14.57%. Found C, 51.74; H, 4.02; N, 10.02; S, 14.18%. IR (KBr) ν_max_/cm^−1^: 1437 (ν(thiadiazole)); 1272 (ν_asym_ (S = O)), 1128 (ν_sym_ (S = O)), 927 (ν(S–N)). UV/Vis (solid) λ_max_/nm: 310 (π→π*), 418(LMCT), 598 (d-d). (ε = 100 cm^−1^M^−1^) (Yield ca. 58%).

### 3.2. Synthesis of the Complex [Cu(N-(5-Ethyl--[1,3,4]-Thiadiazole-2-yl) Naphtalenesulfonamidate)_2_(py)_2_(H_2_O)] (C2)

A solution containing 2 mmols CuSO_4_· 5H_2_O in 100 mL of pyridine: H_2_O [v:v = 1:1] was added dropwise to 1 mmol of the sulfonamide ligand (HL2) dissolved in 25 mL pyridine: H_2_O [v:v = 2:3], under continuous stirring. The resulting solution was stirred at room temperature for four hours and left to stand at room temperature. After two weeks, by slow evaporation of the solvent, blue crystals suitable for X-ray diffraction were obtained.

Anal. Calcd for **(C2)** C_38_H_35_CuN_8_S_4_O_5_ (MW = 875.52 g mol^−1^): C, 52.08; H, 3.99; N, 12.79; S, 14.62%. Found C, 51.92; H, 4.15; N, 12.82; S, 14.03%. IR (KBr) ν_max_/cm^−1^: 1450 (ν(thiadiazoleS)); 1268 (ν_asym_ (S = O)), 1125 (ν_sym_ (S = O)), 932 (ν(S–N)). UV/Vis (solid) λ_max_/nm: 304 (π→π*), 423 (LMCT), 587 (d-d). (Ɛ = 94 cm^−1^M^−1^) (Yield ca. 62%).

### 3.3. X-ray Single Crystal Diffraction and Structures Refinement

Single crystals of two cooper complexes were attached on a nylon loop and mounted on the goniometer of a SuperNova diffractometer, which was equipped with two micro-sources (Mo and Cu), Eos CCD detector, and X-ray tube, set at 50 kV and 0.8 mA. Experimental data were collected and corrected by Lorentz, polarization, and absorption effects in CrysAlis PRO [51]. The crystal structures of the complexes were solved with SHELXT [52] using Intrinsic Phasing and were refined via the SHELXL [53] refinement package using least squares minimization, all being implemented in Olex2 software [54]. Carbon-bound hydrogens were located, refined, and treated using a standard riding procedure, considering the isotropic displacement parameter U_iso_(H) = 1.2U_eq_(C) for ternary CH groups [C-H = 0.93 Å] and secondary CH_2_ groups [C-H = 0.97 Å] and 1.5U_eq_(C) considered for all methyl CH_3_ groups [C-H = 0.96 Å]. Oxygen-bound hydrogen atoms were geometrically located and refined as riding.

### 3.4. X-ray Powder Diffraction

The copper complexes’ diffraction patterns were recorded with a Bruker D8 Advance diffractometer (40 kV, 40 mA) using monochromatic CuKα1 radiation (λ = 1.54056 Å) with a Ge (111) monochromator and equipped with a LYNXEYE detector. A scanning rate of 0.01 °/s was employed for data acquisition with the DIFFRAC plus XRD Commander program.

### 3.5. 3D Hirshfeld Surfaces and Related Fingerprint Plots Analysis

Based on CIF files, Hirshfeld 3D surfaces and the corresponding 2D fingerprint diagrams were obtained by mapping the d_norm_ function [55]. The calculations were performed using CrystalExplorer software [56] and the C-H and O-H bond lengths normalized to known values of neutron diffraction.

### 3.6. In Vitro Biological Assays

#### 3.6.1. DNA Cleavage

Reactions were performed by mixing 7 μL of cacodylate buffer 0.1 M, pH 6 (cacodylic acid/sodium cacodylate), 6 μL of complex solution (final concentrations: 5, 10, 15, and 20 μM), 1 μL of pUC18 DNA solution (0.25 μg/μL, 750 μM in base pairs), and 6 μL of activating agent solution (H_2_O_2_/ascorbic acid) in a 3-fold molar excess relative to the concentration of the complex. The resulting solutions were incubated for 1 h at 37 °C, after which a quench buffer solution (3 μL) consisting of bromophenol blue (0.25%), xylene cyanol (0.25%), and glycerol (30%) was added. The solution was then subjected to electrophoresis on 0.8% agarose gel in 0.5 × TBE buffer (0.045 M Tris, 0.045 M boric acid, and 1 mM EDTA) containing 5 μL/100 mL of a solution of ethidium bromide (10 mg/mL) at 100 V for 2 h. The bands were photographed on a capturing system (Gelprinter Plus TDI) [57].

To test for the presence of reactive oxygen species (ROS) generated during strand scission and for possible complex–DNA interaction sites, various reactive oxygen intermediate scavengers and groove binders were added to the reaction mixtures. The scavengers used were 2,2,6,6-tetramethyl-4-piperidone (0.5 M), dimethylsulfoxide (DMSO) 14 M, *t*-butyl alcohol (10.5 M), sodium azide (NaN_3_) (400 mM), and superoxide dismutase (SOD) (15 units). In addition, a chelating agent of copper(I) and neocuproine (36 μM), along with the groove binder distamycin (80 μM) were also assayed. Samples were treated as described above.

#### 3.6.2. SOD Mimetic Activity

The in vitro SOD mimetic activities of the complexes were assayed using the Oberley and Spitz method with some minor modifications [58]. To generate a reproductible and constant flux of superoxide anions, xanthine (1.5 × 10^−4^ M) and xanthine oxidase in 50 mM potassium phosphate buffer, pH = 7.8, were used. The superoxide anions were detected by the reduction of nitro blue tetrazolium (NBT) (5.6 × 10^−5^ M) to blue formazane, which was determined spectrophotometrically at 560 nm. Different concentrations of the complexes (50, 25, 12.5, 2, and 0.2 μM) were prepared in 50 mM Tris-HCl buffer, pH = 7.8. For each determination, 0.1 mL complex solution + 0.1 mL xanthine oxidase were added to 0.8 mL of solution containing 0.69 mL potassium phosphate buffer (pH = 7.8), 0.025 mL NBT, and 0.085 mL xanthine. The inhibition percentage of NBT reduction was used to determine the SOD activities of the complexes. The conversion of xanthine to uric acid was measured at 310 nm. The % inhibition of enzyme activity was subtracted from that of NBT. The IC_50_ values (the concentration of complex needed to yield 50% inhibition of NBT reduction) were determined from plots of % inhibition versus complex concentration. A Specord 200 Plus Spectrophotometer was used to perform the determinations and all reagents were purchased from Sigma-Aldrich.

#### 3.6.3. Cell Culture and Cytotoxicity Assays

##### Cell Culture

To determine the cytotoxicity of the copper complexes, three cell lines were used: a human cervical carcinoma line (HeLa), a human radical growth phase melanoma cell line (WM35), and a normal fibroblastic epithelial cell line (HFL1). The cell lines were obtained from ATCC (American Type Cell Collection, Manassas, VA 20110-2209, USA) and were maintained in DMEM (Sigma-Aldrich, Saint Louis, Missouri, USA) supplemented with 10% fetal bovine serum (Hyclone), 1 m*M* glutamine (Sigma-Aldrich), and 1% antibiotic antimycotic 100× (Sigma-Aldrich). The cells were cultured at 37 °C in an atmosphere of 5% CO_2_ and 95% relative humidity [59,60].

##### Cytotoxicity Assays

The cytotoxicity assays were performed using the MTT assay (3-(4,5-dimethylthiazol-2-yl)-2,5-diphenyl tetrazolium bromide-Sigma Aldrich). Cells were plated at a density of 1 × 10^5^ cells/well in 96-well plates for 24 h in normal propagation media. The cultures were then exposed to **C1** and **C2** complexes in different concentrations (50, 25, 12.5, 2, and 0.2 µM). The complexes were first solubilized in DMSO (Sigma) and then serial dilutions in media were performed until the final concentration of DMSO was less than 1%. As a positive control, cisplatin (Ebewe Pharma Ges.m.b.H. Nfg. KG, Austria) was used, in the same concentrations as the complexes. As a negative control, cell lines cultivated in normal expansion medium with the same amount of DMSO added were used. The cellular viability was determined using the MTT assay at 24, 48, and 72 h, respectively. Formazan particles were solubilized with DMSO and absorbances were determined at 550 nm using a microplate reader (Bio-Rad, Hercules, CA, USA). The cytotoxic activity, expressed as IC_50_ values representing the complex solution required to inhibit 50% of the cell proliferation, were calculated from the calibration curve by nonlinear regression. Each experiment was carried out in triplicate [61,62].

#### 3.6.4. Evaluation of Antibacterial Activity

The disk diffusion method [63,64,65] was used to determine the antibacterial activity of the **C1** and **C2** copper complexes. The method was adapted for the purposes of the current study, according to the standards developed by the Clinical and Laboratory Standards Institute [66].

Four reference microbial strains, two Gram-positive (*Staphylococcus aureus* ATCC 6538P and *Bacillus cerreus* ATCC 14579) and two Gram-negative (*Escherichia coli* ATCC 10536 and *Pseudomonas aeruginosa* ATCC 27853) strains, were used for in vitro susceptibility testing. These reference microbial strains were obtained from the American Type Culture Collection (ATCC, Manassas, VA, USA). As positive controls, Amoxicillin (Oxoid Ltd., Basingstoke, UK) and Norfloxacin (Sigma-Aldrich) were used.

For each of the four species, an initial suspension of bacterial cultures was inoculated on nutrient agar plates (Merck KGaA, Darmstadt, Germany), incubated for 24 h at 37 ± 2 °C and resuspended in a physiological saline buffer to a 10^6^ CFU/mL concentration (on a 0.5 McFarland scale), and further inoculated on Muëller Hinton agar plates (Merck KGaA, Darmstadt, Germany). The initial inoculum was similar to that prepared for the classical antibiotic susceptibility test, so the copper complexes’ effects (in terms of sensitivity) were comparable to those of the regular antibiotics (Amoxicillin, Norfloxacin). After inoculation, the medium surface was dried and 8 wells were radially drilled 1.5 cm from the outer edge, 3 cm apart. From aliquot samples of 1 mg/mL of each copper complex dissolved in DMSO and incorporated in sterile liquid medium, 5 µL was placed in each of the 8 wells and left for 30 min to diffuse into the agar plates. The plates were then incubated for 24 h at 37 ± 2 °C. Then, 25 µg Amoxicillin and 5 µg Norfloxacin served as a positive controls, while DMSO 0.75% was used as a negative control [67].

Readings were conducted by measuring the diameter of the inhibition zone (Halo Zone Test, in mm). The germ sensitivity of the copper complexes was estimated by comparing the diameter of the inhibition area to that generated by the regular antibiotics with known inhibition values. As the test compound was more active, the inhibition of the microbial growth was more extensive. All tests were triplicated, and the measured diameters of the inhibition zone were expressed (in mm) as mean ± standard deviation.

To quantify the copper complexes effectiveness as antibacterial agents, their minimum inhibitory concentrations (MICs) were determined using the serial broth microdilution method described by Quinn et al. [68] and Markey et al. [69]. The method was adapted for the purposes of the current study, according to the standards developed by the Clinical and Laboratory Standards Institute [66]. The tests were performed on the same Gram-positive and Gram-negative reference microbial strains. Ten successive dilutions of the copper complexes solutions (1/2 to 1/1024) in nutrient broth (Merck KGaA, Darmstadt, Germany) were performed and then were placed in sterile microplates (one each for every copper complex). The microplates were incubated at 37 ± 2 °C for 24 h. Comparisons of the amount of bacterial growth in each well containing copper complexes solutions with the amount of growth in the growth control wells were performed and the maximal dilution for which the tested copper complexes inhibited bacterial growth was determined.

### 3.7. In Vivo Toxicity Study of C2 Complex

A subacute toxicity study was carried out for a period of 2 weeks for the **C2** complex. In total, 10 adult Swiss mice, 32.26 ± 1.3 g body weight, with 5 males and 5 females, were used. The mice were caged in standard polypropylene cages, in standard laboratory conditions (12 h light/dark cycle, temperature 25 ± 1 °C, and relative humidity 55 ± 5%). Standard lab chow, provided by the “Cantacuzino” National Insitute for Research and Development Bucharest, and water were freely available. All procedures performed on mice complied with the European Directive 2010/63/EU and the national low 43/2014 for Protection of Animals Used for Scientific Purposes. This study was approved by the Bioethical Board of the Faculty of Veterinary Medicine Cluj-Napoca (accord no.290/22.11.2021) and the Veterinary Sanitary Direction and Food Safety (accord no.284/21.12.2021) [70]. All mice received i.p. 150 mg complex **C2**/kg b.w./day, for 2 weeks. The injectable form was obtained by dissolving the complex **C2** crystals in a mixture of glycerol formal and 1,2- propanediol (Sigma-Aldrich) in rapport 2:3 (adapted accordingly to the patent no. A01251/30.11.2010), obtaining a concentration of 100 mg/mL. Before injection, the mixture was further diluted in NaCl 9‰ sterile solution.

The general clinical status and the body weight were recorded daily. For the biochemical and hematological analysis [71], at the end of the 14 days, blood samples were taken from the orbitary sinus under deep narcosis. Then, the mice were euthanized by narcotic overdose.

The liver and kidneys were also removed. Immediately after, gross examination was performed, and fragments of kidney and liver were fixed in buffered formalin and embedded in paraffin wax. Histological analysis of the hepatic and renal tissues was performed using an Optical Microscope Olympus 1 CX41, after the hematoxylin eosin straining was carried out. Concerning the biochemical determinations targeting the exploration of the liver and kidney toxicity, transaminases, albumin, total protein, creatinine, and urea determinations were performed using a STAT-FAX 1904 Plus semi-automatic analyzer and special determination kits. To determine the hematological toxicity, erythrogram, leukogram, and thrombogram were assessed using a Diatron Abascus Junior Vet 3.

### 3.8. Statistical Analysis

All data are reported as the mean ± standard deviation (S.D.). Statistical values were obtained using GraphPad Prism version 5.0 for Windows, GraphPad Software, San Diego, CA, USA. 

## 4. Conclusions

Two Cu^+2^ complexes, [Cu(L1)_2_(H2O)_3_] (C1) and [Cu(L2)_2_(py)_2_(H2O)] (C2) were synthesized and characterized. The crystalline structures of the complexes were determined using X-ray diffraction and were confirmed by the data obtained from elemental analysis, spectral (IR, UV-Vis, EPR), and magnetic determinations. Their “in vitro” nuclease, SOD-mimetic, cytotoxic, and antibacterial activities were demonstrated. The complexes showed superior nuclease activity compared to the non-coordinated Cu^+2^ ion. C1 and C2 complexes presented SOD-like activity but were weaker than native SOD. Both complexes presented cytotoxicity on tumor cell lines (HeLa, WM35) and C2 complex was more active than cisplatin. Both complexes were less toxic on normal fibroblastic cells (HFL1) than cisplatin, which represents a great advantage. Another great finding of this study is that both copper complexes showed antibacterial activity on *Pseudomonas aeruginosa*, which is resistant to Amoxicillin exposure. Both complexes showed in vitro biological activity, but the **C2** complex was more active. A lack of in vivo toxicity was demonstrated for the **C2** complex by performing hepatic, renal, and hematological studies on Swiss mice. Nowadays, the discovery of a new non-toxic metal complex with biological activity represents a very active area of research. In the future, we intend to study the in vivo biological activity of the **C2** complex.

The Supplementary Material has been deposited with the Cambridge Crystallographic Data Centre (nos. 2166573 (C1).

## Figures and Tables

**Table 1:** Crystallographic details of the investigated crystals.

Identification Code	Complex C1	Complex C2
Empirical formula	C_38_H_34_CuN_6_O_7_S_4_	C_38_H_36_CuN_8_O_5_S_4_
Formula weight	878.49	876.53
Temperature/K	293(2)	293(2)
Crystal system	monoclinic	monoclinic
Space group	P2_1_/c	C2/c
a/Å	5.6797(2)	24.6386(5)
b/Å	42.8168(16)	9.3411(2)
c/Å	17.2267(5)	18.5012(4)
α/°	90	90
β/°	92.742(3)	108.467(2)
γ/°	90	90
Volume/Å^3^	4184.5(2)	4038.82(15)
Z	4	4
ρ_calc_g/cm^3^	1.394	1.442
μ/mm^−1^	3.055	3.139
F (000)	1812.0	1812.0
Crystal size/mm^3^	0.11× 0.10 × 0.07	0.11 × 0.10 × 0.09
Radiation	CuKα (λ = 1.54184)	CuKα (λ = 1.54184)
2Θ range for data collection/°	8.048 to 141.334	7.566 to 140.94
Index ranges	−6 ≤ h ≤ 4, −51 ≤ k ≤ 49, −20 ≤ l ≤ 21	−29 ≤ h ≤ 26, −11 ≤ k ≤ 5, −22 ≤ l ≤ 21
Reflections collected	15281	7314
Independent reflections	7828 [R_int_ = 0.0350, R_sigma_ = 0.0458]	3784 [R_int_ = 0.0351, R_sigma_ = 0.0344]
Data/restraints/parameters	7828/0/510	3784/2/264
Goodness-of-fit on F^2^	1.061	1.106
Final R indexes [I ≥ 2σ (I)]	R_1_ = 0.0784, wR_2_ = 0.2281	R_1_ = 0.0592, wR_2_ = 0.1521
Final R indexes [all data]	R_1_ = 0.0894, wR_2_ = 0.2403	R_1_ = 0.0614, wR_2_ = 0.1547
Largest diff. peak/hole/e Å^−3^	1.19/−1.27	0.65/−1.40

**Table 2:** IC_50_ values of the complexes **C1** and **C2** and cisplatin on the human cervical carcinoma line (HeLa), human radical growth phase melanoma cell line (WM35), and normal fibroblastic epithelial cell line (HFL1) (versus untreated cells) (mean ± SD) (n = 3).

Cells	Complex	IC50 (µM)		
		24 h	48 h	72 h
HeLa cells	**C1**	33.18 ± 0.19	16.36 ± 0.12	6.47 ± 0.06
	**C2**	8.79 ± 0.21	4.06 ± 0.05	1.45 ± 0.09
	Cisplatin	21.03 ± 0.14	6.02 ± 0.19	2.39 ± 0.04
WM35 cells	**C1**	41.35 ± 0.19	23.87 ± 0.17	15.42 ± 0.08
	**C2**	13.01 ± 0.15	8.11 ± 0.13	4.66 ± 0.07
	Cisplatin	26.07 ± 0.43	11.15 ± 0.09	5.98 ± 0.03
HFL1 cells	**C1**	44.67 ± 0.51	7.38 ± 0.12	3.75 ± 0.15
	**C2**	17.99 ± 1.08	5.55 ± 0.22	2.03 ± 0.04
	Cisplatin	13.22 ± 0.89	3.99 ± 0.22	1.18 ± 0.13

**Table 3:** Evaluation of the antibacterial activity using the disk diffusion method. Effect of copper complexes on bacterial strains *(Halo Zone Test/mm)* (mean ± SD) (n = 3).

Bacterial Strains	Antibiotics		C1 Complex	C2 Complex	Negative Control
	Amoxicillin	Norfloxacin			
*Staphylococcus aureus* ATCC 6538P	19 ± 0.14	16 ± 0.21	13 ± 0.17	15 ± 0.44	R
*Bacillus cerreus* ATCC 14579	13 ± 0.22	18 ± 0.19	11 ± 0.25	12 ± 0.34	R
*Escherichia coli* ATCC 10536	18 ± 0.09	20 ± 0.11	14 ± 0.11	16 ± 0.21	R
*Pseudomonas aeruginosa* ATCC 27853	R	25 ± 0.24	15 ± 0.32	19 ± 0.19	R

R = resistant.

## Data Availability

The data presented in this study are available within the article.

## Supplementary Materials

The following supporting information can be downloaded at: https://www.mdpi.com/article/10.3390/molecules27103338/s1, Figure S1: IR spectrum of the complex **C1**; Figure S2: IR spectrum of the complex **C2**; Figure S3: UV-VIS spectrum of the complex **C1**; Figure S4: UV-VIS spectrum of the complex **C2**; Figure S5: RES spectrum of the complex **C1**; Figure S6: RES spectrum of the complex **C2**; Figure S7: Electroferogram in agarose gel of the pUC18 plasmid treated with CuSO_4_, the **C1** and **C2** complexes and bis(o-phenathroline) Cu^+2^; Figure S8: Electroferogram in agarose gel of the pUC18 plasmid treated with the **C1** and **C2** complexes and various inhibiting agents; Figure S9: Histological analyses of renal cortical and hepatic lobe; Table S1: Selected bond lengths and angles for the complex **C1**; Table S2: Selected bond lengths and angles for complex **C2**; Table S3: Hydrogen bonds geometries in investigated crystals; Table S4: Minimum inhibitory concentration of copper complexes; Table S5: The influence of complex **C2** on biochemical parameters; Table S6: The influence of complex **C2** on erythrogram; Table S7: The influence of complex **C2** on leukogram; Table S8: The influence of complex **C2** on thrombogram.

## Sample Availability

Samples of the compounds are available on request from the corresponding author.
