# Supplementary material for: New Cu+2 Complexes with N-Sulfonamide Ligands: Potential Antitumor, Antibacterial, and Antioxidant Agents
Source: Molecules. 2022 May 23;27(10):3338. doi: 10.3390/molecules27103338 (PMC9144936; doi:10.3390/molecules27103338)
Supplement: Supplementary file 1 [file molecules-27-03338-s001.zip › molecules-1733301-SI.pdf]

Supplementary materials

New Cu^{+2} Complexes with N-Sulfonamide Ligands: Potential Antitumor, Antibacterial, and Antioxidant Agents

Adriana Corina Hangan [1], Alexandru Turza [2], Roxana Liana Lucaciu [3,*], Bogdan Sevastre [4], Emőke Páll [4], Luminița Simona Oprean [1] and Gheorghe Borodi [2]

1 Department of Inorganic Chemistry, Faculty of Pharmacy, "Iuliu-Hațieganu" University of Medicine and Pharmacy, 400012 Cluj-Napoca, Romania; acomsa6@yahoo.com (A.C.H.); loprean@umfcluj.ro (L.S.O.)
2 National Institute for Research and Development of Isotopic and Molecular Technologies, 400293 Cluj-Napoca, Romania; turzaalex@yahoo.com (A.T.); borodi@itim-cj.ro (G.B.)
3 Department of Pharmaceutical Biochemistry and Clinical Laboratory, Faculty of Pharmacy, "Iuliu-Hațieganu" University of Medicine and Pharmacy, 400012 Cluj-Napoca, Romania
4 Paraclinic/Clinic Department, Faculty of Veterinary Madicine, University of Agricultural Science and Veterinary Medicine, 400372 Cluj-Napoca, Romania; bogdan.sevastre@usamvcluj.ro (B.S.); emoke.pall@usamvcluj.ro (E.P.)
* Correspondence: roxanaluc@yahoo.com

Table S1. Selected bond lengths (Å) and angles (°) for the complex **C1.**

Bond	d (Å)	Bond	d (Å)	Angle	(°)	Angle	(°)
N2A-C11A (thiadiazole)	1.315	N2B-C11B (thiadiazole)	1.323	N1A-S1A-O2A	105.98	N1B-S1B-O2B	107.30
N3A-C12A (thiadiazole)	1.299	N3B-C12B (thiadiazole)	1.297	N1A-S1A-O1A	112.73	N1B-S1B-O1B	112.76
C11A-S2A (thiadiazole)	1.752	C11B-S2B (thiadiazole)	1.745	N1A-S1A-C8A	105.01	N1A-S1A-C8A	106.73
C12A-S2A (thiadiazole)	1.736	C12B-S2B (thiadiazole)	1.731	O2A-S1A-C8A	107.52	O2B-S1B-C8B	105.94
N2A-N3A (thiadiazole)	1.380	N2B-N3B (thiadiazole)	1.388	O1A-S1A-C8A	107.36	O1A-S1A-C8A	106.08
N1A-C11A	1.355	N1B-C11B	1.342	O1A-S1A-O2A	117.46	O1B-S1B-O2B	117.32
N1A-S1A	1.602	N1B-S1B	1.601	C11A-N1A-S1A	120.67	C11A-N1A-S1A	121.99
S1A-O2A	1.441	S1B-O2B	1.437	N2A-C11A-N1A	120.62	N2B-C11B-N1B	120.40
S1A-O1A	1.452	S1B-O1B	1.441	S1A-C11A-N1A	128.14	S1B-C11B-N1B	128.24
S1A-C8A	1.759	S1B-C8B	1.775	N2A-Cu1-O1W	91.14	N2B-Cu1-O1W	92.20

Cu1-N2A	2.012	Cu1-N2B	2.013	N2A-Cu1-O3W	88.99	N2B-Cu1-O3W	87.89
Cu1-O1W	2.002			O2W-Cu1-O3W	92.09	O2W-Cu1-O1W	86.61
Cu1-O2W	2.435						
Cu1-O3W	1.982						

Table S2. Selected bond lengths (Å) and angles (°) for complex **C2**.

Bond	d (Å)	Angle	(°)
N2-C11 (thiadiazole)	1.323	N1-S1-O2	113.10
N3-C12 (thiadiazole)	1.273	N1-S1-O1	106.52
C11-S2 (thiadiazole)	1.757	N1-S1-C8	106.04
C12-S2 (thiadiazole)	1.741	O2-S1-C8	107.06
N2-N3 (thiadiazole)	1.386	O1-S1-C8	106.75
N1-C11	1.332	O1-S1-O2	116.71
N1-S1	1.590	C11-N1-S1	120.62
S1-O2	1.444	N2-C11-N1	120.86
S1-O1	1.447	S1-C11-N1	129.05
S1-C8	1.770	N2-Cu1-O1W	96.87
Cu1-N2	1.987	N2-Cu1-N4	86.81
Cu1-O1W	2.285	O1W-Cu1-N4	91.67
Cu1-N4	2.064		

Table S3. Hydrogen bonds geometries in investigated crystals (Å, °).

Structure	D-H···A	D-H	H···A	D···A	<(D-H···A)
Complex **C1**	O1W-H1WB···O2B	0.872	2.135(4)	2.965(3)	158.8(8)
	O2W-H2WB···N1A	0.858	2.163(2)	2.893(1)	142.8(3)
	O2W-H2WA···N1B	0.859	2.219(1)	2.927(3)	139.6(1)
	C3B-H3B···O1B	0.930	2.498(1)	3.317(2)	147.0(5)
	C2B-H2B···S2B	0.930	2.826(4)	3.722(1)	161.9(5)
	C19-H19B···O1A	0.960	2.512(3)	3.457(6)	168.5(6)
Complex **C2**	O1W-H1WA···O1	0.850	2.118(4)	2.950(1)	165.7(8)
	O2D-H2D···O1B	0.970	2.546(6)	4.773(4)	143.9(1)

Table S4: Minimum inhibitory concentration of copper complexes.

Bacterial strains	MIC (mg/l)	
	**C1 complex**	**C2 complex**
Staphylococcus aureus ATCC 6538P	0.289	0.202
Bacillus cerreus ATCC 14579	0.304	0.293
Escherichia coli ATCC 10536	0.231	0.184
Pseudomonas aeruginosa ATCC 27853	0.196	0.089

Table S5. The influence of complex **C2** on biochemical parameters

**Group**	**ALAT** (U/L)	**ASAT** (U/L)	**Total protein** (g/dL)	**Albumin** (g/dL)	**Creatinine** (mg/dL)	**Urea** (mg/dL)
Male	44.27±1.25	169.48±3.66	5.88±0.31	3.29±0.42	0.56±0.12	19.36±2.12
Female	56.49±2.18	195.29±4.21	6.12±0.14	3.87±0.55	0.67±0.15	21.27±3.23

Normal values: ALAT 26-77U/L, ASAT 54-269 U/L, Total protein 3.5-7.2g/dL, Albumin 2.5-4.8g/dL, Creatinine 0.3-1 mg/dL, Urea 12-28 mg/dL (Mean±S.E.M.) (n=5) [48]

Table S6. The influence of complex **C2** on erythrogram

Group	RBC (10^{12}/L)	HGB (g/dL)	HCT (%)	MCH (pg)	MCHC (g/dL)	MCV (fl)	RDWs (fl)
Male	9.19±0.63	13.62±1.022	43.28±2.01	16.25±0.17	32.86±0.49	47.26±1.99	34.37±1.03
Female	9.54±1.03	14.71±1.21	45.06±2.88	16.78±0.76	32.83±0.55	48.98±2.63	36.29±1.25

RBC: red blood cells; HGB: haemoglobin; HCT: hematocrit; MCH: mean corpuscular haemoglobin; MCHC: mean corpuscular haemoglobin concentration; MCV: mean corpuscular volume; RDW: red blood cells distribution width; Normal values: RBC 7-12.5 10^{12}/L, HGB 10.2-16.6 g/dL, HCT 39-49 % (Mean±S.E.M.) (n=5) [48]

Table S7. The influence of complex **C2** on leukogram

Group	WBC (10^9/L)	LYM (10^9/L)	GRA (10^9/L)	MID (10^9/L)
Male	9.67±1.21	6.51±0.99	2.42±0.89	0.29±0.07
Female	10.55±1.01	6.98±0.76	2.82±1.03	0.33±0.15

WBC: white blood cells; LYM: lymphocytes; GRA: granulocytes; MID: middle cells; Normal value: WBC 6-15 10^9/L (Mean±S.E.M.) (n=5) [48]

Table S8. The influence of complex **C2** on thrombogram

Group	PLT (10^6/L)	PCT (%)	MPV (fl)	PDWs (fl)
Male	922.20±100.11	0.62±0.12	6.78±0.34	7.33±0.19
Female	944.96±136.39	0.75±0.24	7.59±0.82	8.99±1.06

PLT: total platelet count; PCT: platelet hematocrit; MPV: mean platelet volume; PDWs: platelet distribution width; Normal value: PLT 800-1100 10^6/L (Mean±S.E.M.)(n=5) [48]

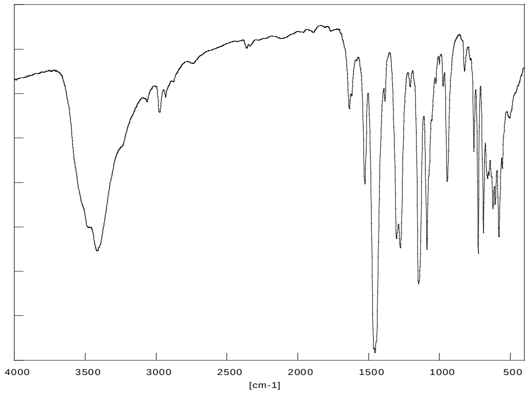

Figure S1. IR spectrum of the complec **C1**

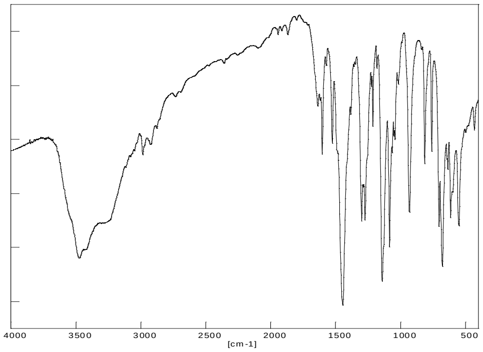

Figure S2. IR spectrum of the complex **C2**

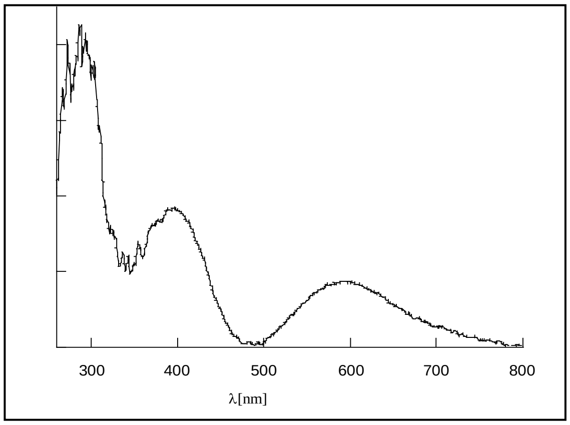

Figure S3. UV-VIS spectrum of the complex **C1**

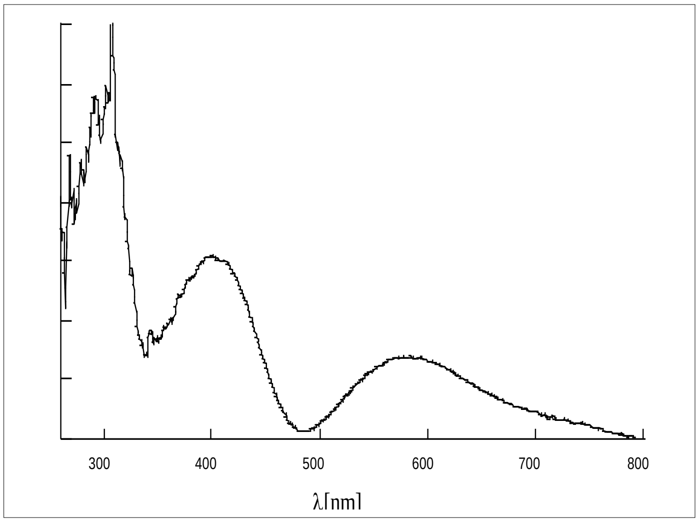

Figure S4. UV-VIS spectrum of the complex **C2**

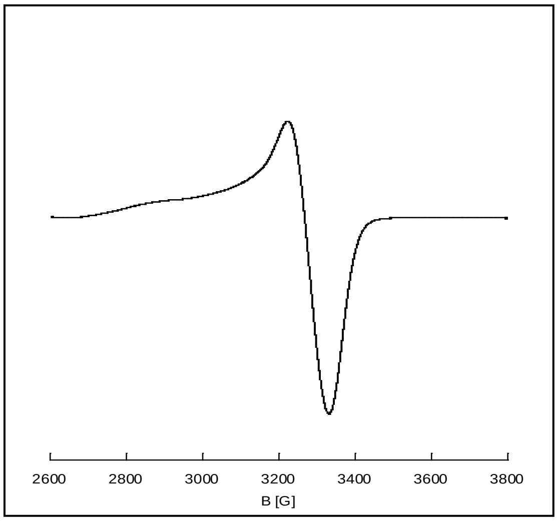

Figure S5. RES spectrum of the complex **C1**

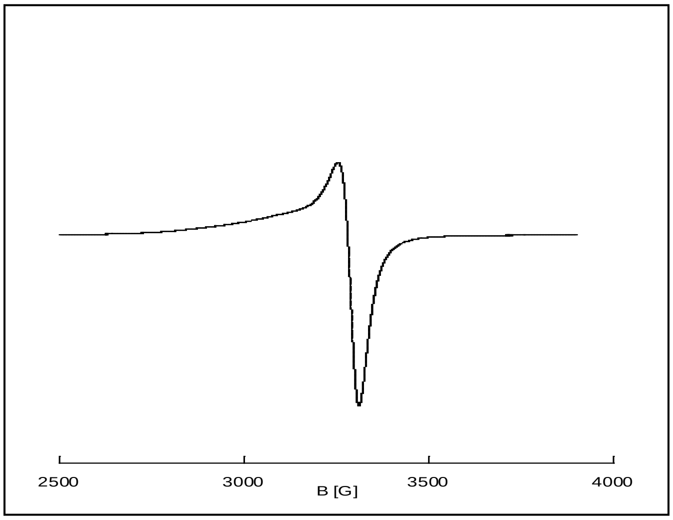

Figure S6. RES spectrum of the complex **C2**

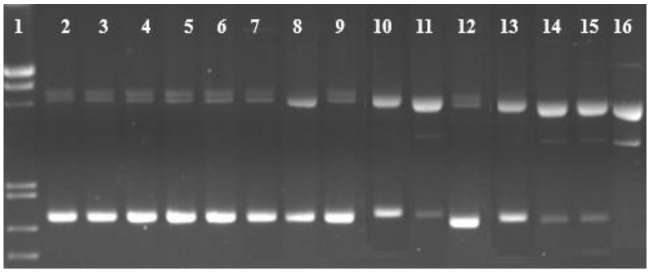

Figure S7. Electroferogram in agarose gel of the pUC18 plasmid treated with $CuSO_4$, the **C1** and **C2** complexes and bis(o-phenathroline) copper(II).
1.base marker; 2.control; 3.control+red.; 4.5μM $CuSO_4$+red.; 5.10μM $CuSO_4$+red.; 6.15μM $CuSO_4$+ red.; 7.20μM $CuSO_4$+red.; 8. 5μM **C1**+red.; 9.10μM **C1**+red.; 10.15μM **C1**+red.; 11.20μM **C1**+red.; 12. 5μM **C2**+red.; 13.10 μM **C2**+red.; 14.15 μM **C2**+red.; 15.20μM **C2**+red.; 16.20μM $[Cu(phen)_2]^{2+}$+ red.

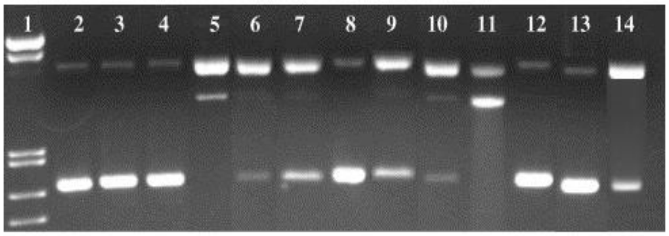

Figure S8. Electroferogram in agarose gel of the pUC18 plasmid treated with the C1 and C2 complexes and various inhibiting agents.
1.base marker; 2. control; 3. complex **C1** 10μM without inhibitors+ reducing agents ; 4. complex **C2** 10μM without inhibitors+ reducing agents; 5. **C1** + DMSO+ reducing agents; 6. **C1** + SOD + reducing agents; 7. **C1** + distamycin + reducing agents; 8. **C1** + neocuproine + reducing agents; 9. **C1 +** NaN_3 + reducing agents; 10. **C2** + DMSO+ reducing agents; 11. **C2** + SOD + reducing agents; 12. **C2** + distamycin + reducing agents; 13. **C2** + neocuproine + reducing agents; 14. **C2 +** NaN_3 + reducing agents

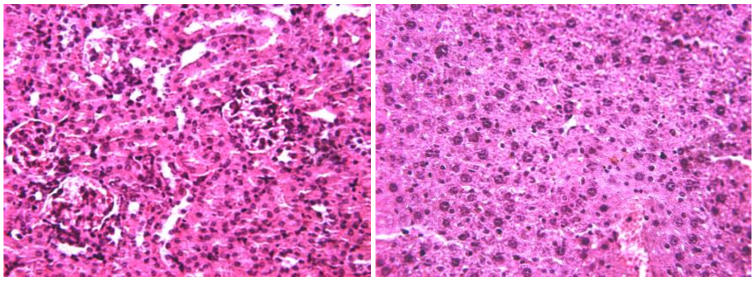

Figure S9. Histological analyses of renal cortical (left) and hepatic lobe (right). HE (x200)
